# Author Correction: Combining different CRISPR nucleases for simultaneous knock-in and base editing prevents translocations in multiplex-edited CAR T cells

**DOI:** 10.1186/s13059-025-03548-z

**Published:** 2025-03-26

**Authors:** Viktor Glaser, Christian Flugel, Jonas Kath, Weijie Du, Vanessa Drosdek, Clemens Franke, Maik Stein, Axel Pruß, Michael Schmueck-Henneresse, Hans-Dieter Volk, Petra Reinke, Dimitrios L. Wagner

**Affiliations:** 1 Berlin Center for Advanced Therapies (Becat), Charité – Universitätsmedizin Berlin, Corporate Member of Freie Universität Berlin and Humboldt-Universität Zu Berlin, Campus Virchow Klinikum, Augustenburger Platz 1, Berlin, 13353 Germany; 2 BIH Center for Regenerative Therapies (BCRT), Berlin Institute of Health at Charité – Universitätsmedizin Berlin, Campus Virchow Klinikum, Augustenburger Platz 1, Berlin, 13353 Germany; 3 Institute of Transfusion Medicine, Charité – Universitätsmedizin Berlin, corporate member of Freie Universität Berlin and Humboldt-Universität zu Berlin, Campus Charité Mitte, Charitéplatz 1, Berlin, 10117 Germany; 4 Institute of Medical Immunology, Charité – Universitätsmedizin Berlin, Corporate Member of Freie Universität Berlin and Humboldt-Universität Zu Berlin, Campus Virchow Klinikum, Augustenburger Platz 1, Berlin, 13353 Germany; 5 CheckImmune GmbH, Campus Virchow Klinikum, Augustenburger Platz 1, Berlin, 13353 Germany

**Correction: Genome Biol 24, 89 (2023)**

**https://doi.org/10.1186/s13059-023–02928-7**

Following publication of the original article [1], the authors identified an error in one of the guide RNA spacer sequences disclosed in Supplementary Table S3. The correct sequence for base editing mediated silencing of the *CIITA* is 5’−3’: CACTCACCTTAGCCTGAGCA, as originally described in Gaudelli et al. 2020 [2].

This error does not affect the main results and conclusions of the paper.

The Supplementary Table S3 of the original article [1] has been corrected.

## Supplementary Information

Additional file 2: Supplementary Tables
