# Supplementary material for: Combining different CRISPR nucleases for simultaneous knock-in and base editing prevents translocations in multiplex-edited CAR T cells
Source: Genome Biol. 2023 Apr 24;24:89. doi: 10.1186/s13059-023-02928-7 (PMC10123993; doi:10.1186/s13059-023-02928-7)
Supplement: Supplementary file 1 — Additional file 1: Fig. S1. Sequencing confirmsefficient base editing at B2M and CIITA after transfection of Cas9 adeninebase editor mRNA. Fig. S2. Comparison of surface expression of CAR, TCR and MHC expression in different multiplex edited T cells. Fig. S3. Co-delivery of modified mRNA for base editing does not reduce T cell viability and expansion capacity during non-viral knock-in. Fig. S4. CRISPResso2 results after amplicon sequencing of CIITA and B2M. Fig. S5. MHC silencing prevents allo-specific T cell cytotoxicity. Fig. S6. High background in ddPCR assay with probe binding TRAC HDRT removed by placing the probe on non-TRAC locus of translocation. [file 13059_2023_2928_MOESM1_ESM.docx]

**Supplementary Figures:**

**Fig. S1: Sequencing confirms efficient base editing at *B2M* and *CIITA* after transfection of Cas9 adenine base editor mRNA**

**
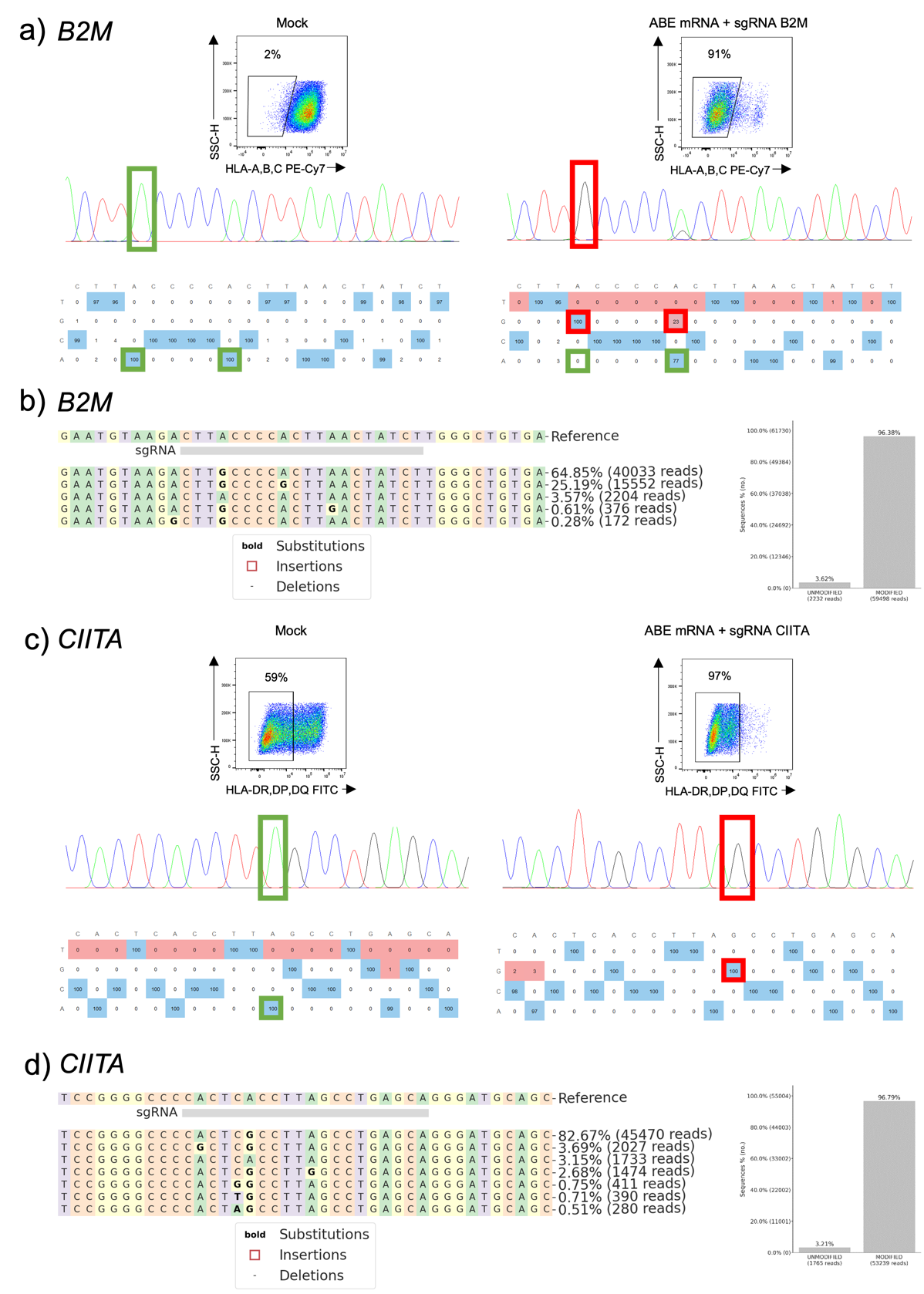
**

**Fig. S1: Sequencing confirms efficient base editing at *B2M* and *CIITA* after transfection of Cas9 adenine base editor mRNA.** T cells were mock-electroporated or transfected with mRNA encoding for the adenine base editor ABE8.20-m with a gRNA targeting a splice site of *B2M* or *CIITA*. Analysis was performed 4 days after transfection. (a+c) Representative flow cytometry and EditR results from Sanger sequencing data at *B2M* locus (a) or *CIITA* locus (c). b+d) Representative results from targeted next generation sequencing confirms high rate of A to G conversions in modified, but not unmodified cells (left: top 5 reads, right: summary of modified reads according to CRISPResso2 analysis) at *B2M* (b) and *CIITA* (d) locus). n=3 healthy donors.

**Fig. S2: Comparison of surface expression of CAR, TCR and MHC expression in different multiplex edited T cells**

**
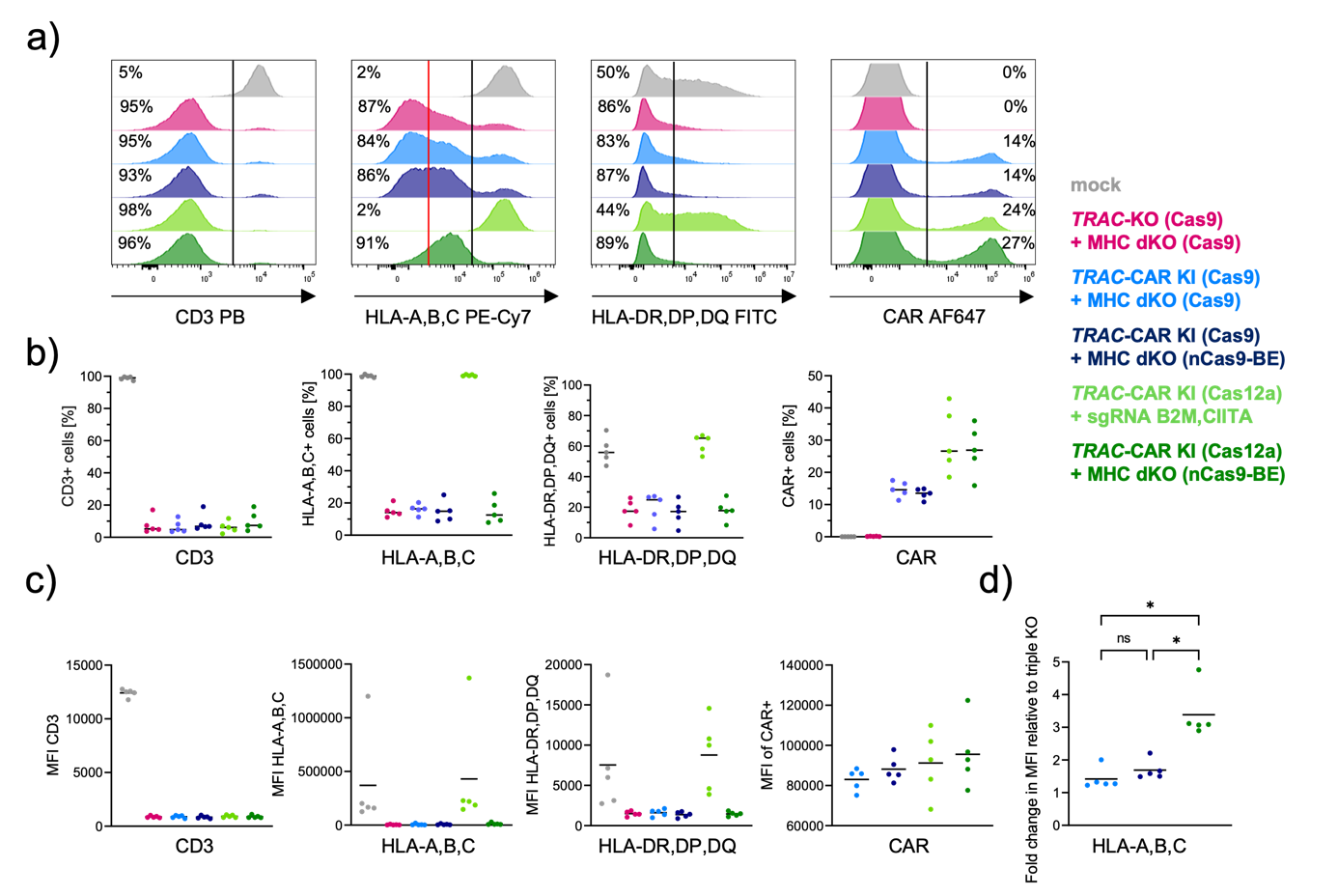
**

**Fig. S2: Comparison of surface expression of CAR, TCR and MHC expression in different multiplex edited T cells.** a) Representative flow cytometry histograms for surface expression of CD3, HLA-A,B,C, HLA-DR,DP,DQ or CAR (aFC) four days after T cells were transfected with or without gene editors. b) Paired summary data for surface marker expression from T cells of three independent donors four days after treatment. c) Summary data of median fluorescent intensity (MFI) for the respective markers after gene editing. d) Normalized MFI data comparing the relative expression level of edited fraction (left of black line in histograms in a) of *TRAC*-KO (Cas9) + MHC dKO (Cas9) cells with different gene edited T cell populations. n=5 healthy donors (independent experiments). Statistical analysis of flow cytometry and ddPCR data from 5 donors was performed using a one-way ANOVA of matched data with Geisser-Greenhouse correction. Multiple comparisons were performed by comparing the mean of each column with the mean of every other column and corrected by the Turkey test. Asterisks represent different p-values calculated in the respective statistical tests (ns : p ≥ 0.05; * : p < 0.05; ** : p < 0.01; *** : p < 0.001; **** : p < 0.0001).

**Fig. S3: Co-delivery of modified mRNA for base editing does not reduce T cell viability and expansion capacity during non-viral knock-in**

**
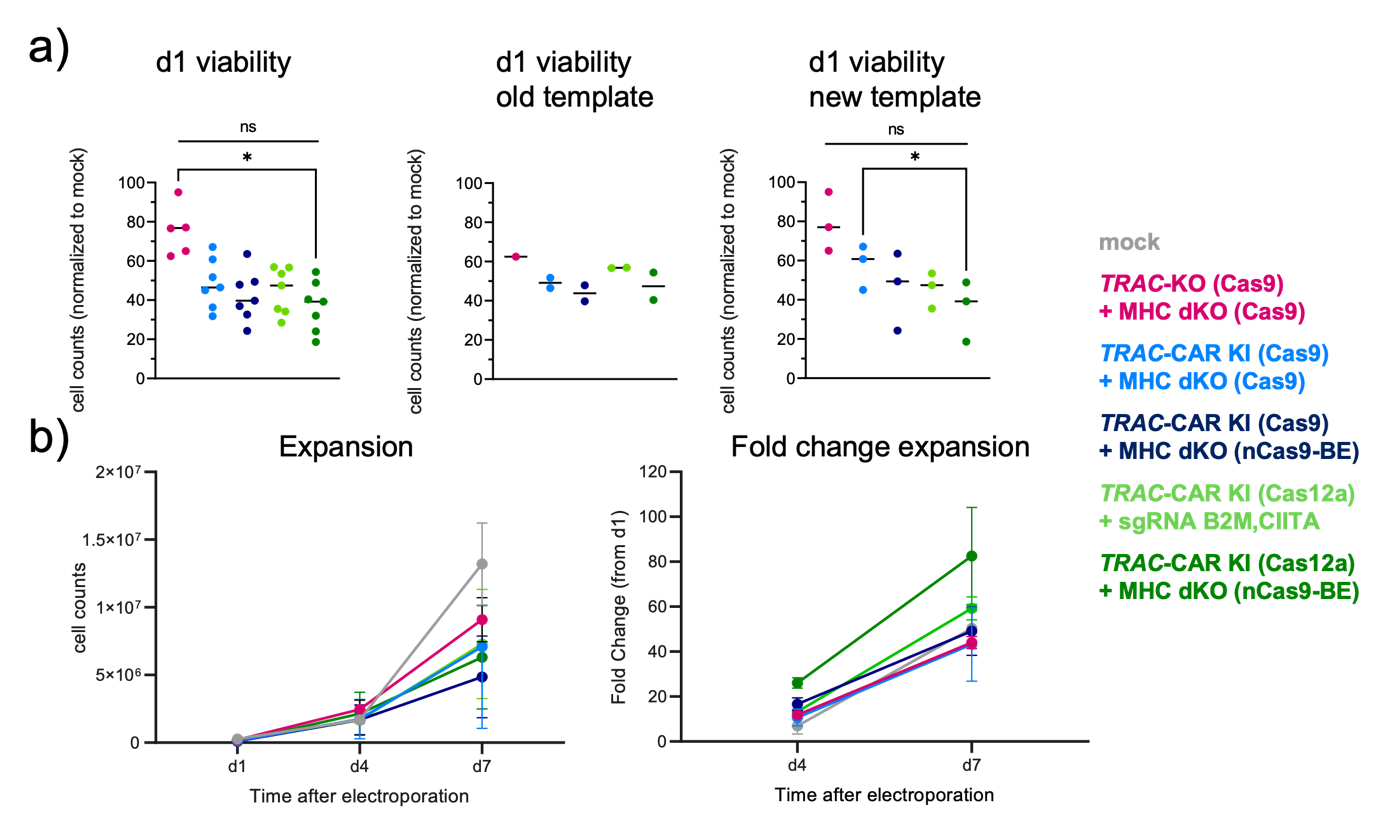
**

**Fig. S3: Co-delivery of modified mRNA for base editing does not reduce T cell viability and expansion capacity during non-viral knock-in.** a) Summary results of cell viability determined 24 hours after transfection and normalized to mock-electroporated T cells. Followed by individual viability results depending on which HDRT template was used (new: PAM-mutated template as displayed in Figure 3). b) Total cell counts within 7 days after nucleofection and summary of fold expansion for different gene-edited T cells normalized to cell counts from 24 hours after transfection (n=2). Statistical analysis of flow cytometry and ddPCR data from 5 donors was performed using a one-way ANOVA of matched data with Geisser-Greenhouse correction. Multiple comparisons were performed by comparing the mean of each column with the mean of every other column and corrected by the Turkey test. Asterisks represent different p-values calculated in the respective statistical tests (ns : p ≥ 0.05; * : p < 0.05; ** : p < 0.01; *** : p < 0.001; **** : p < 0.0001).

**Fig. S4: CRISPResso2 results after amplicon sequencing of *CIITA and B2M***


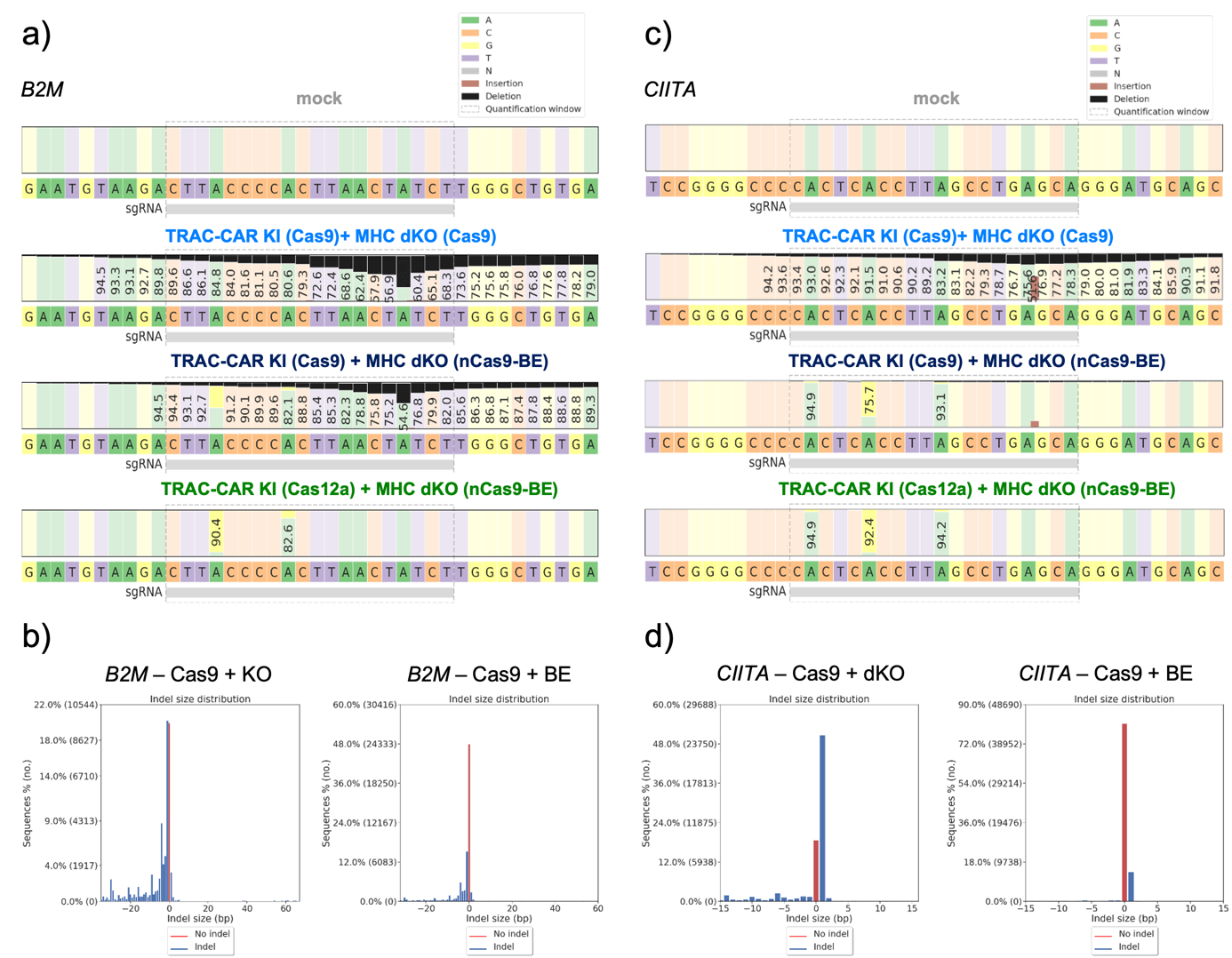


**Fig. S4: CRISPResso2 results after amplicon sequencing of *CIITA and B2M.*** Representative CRISPResso2 results from amplicon sequencing data and Indel size distribution of the a),b) B2M and c,d) CIITA target site from T cells electroporated with different gene editors as described before. n=5 healthy donors.

**Fig. S5: MHC silencing prevents allo-specific T cell cytotoxicity**

**
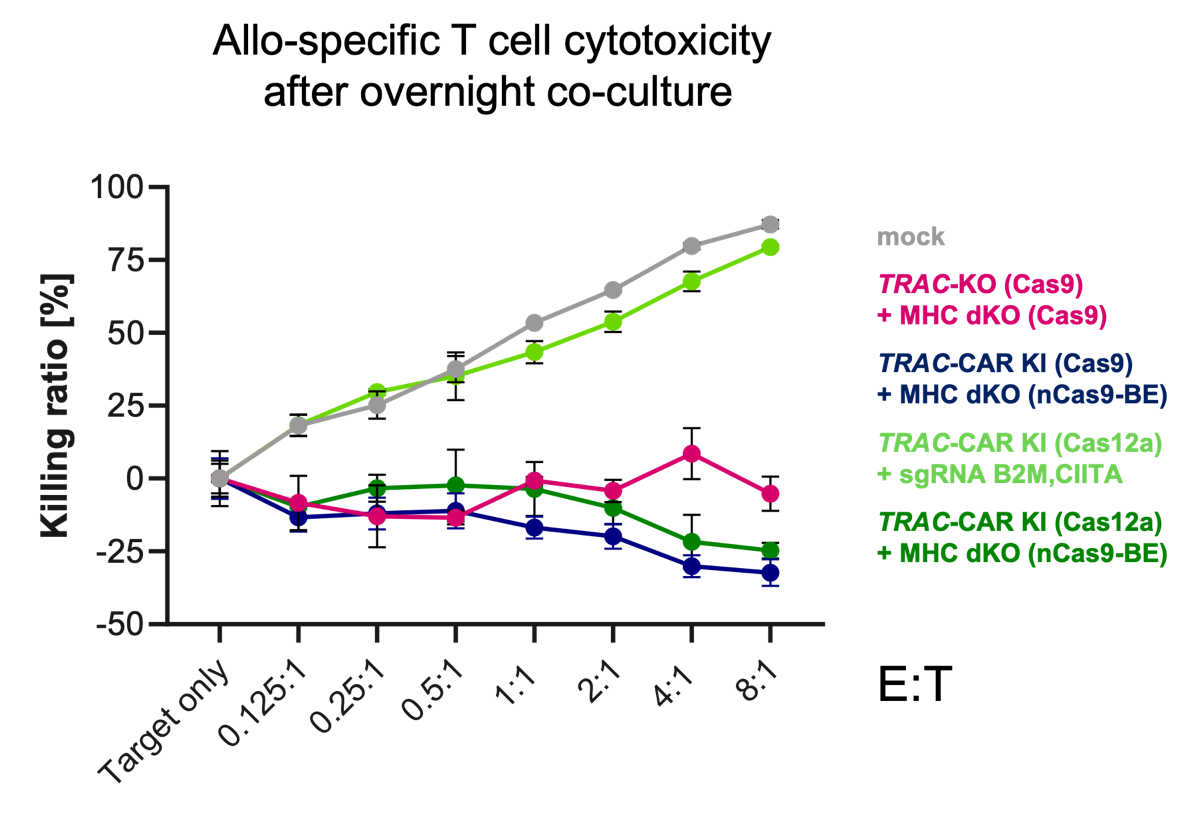
**

**Fig. S5: MHC silencing prevent allo-specific T cell cytotoxicity.** Allo-specific T cells were generated by stimulating NK cell-depleted PBMCs from donor A with irradiated T cell-depleted PBMCs from the donor used for CAR T cell generation. Allo-specific T cells were re-stimulated twice with irradiated T cell-depleted PBMCs from the CAR T cell donor at day 7 and 14 after isolation. Then, allo-specific T cells were used as effector cells during an overnight killing assay. To this end, CAR T cells were labeled with CFSE. Co-cultures were set up at different effector (allo-specific T cells) to target (edited T cells) ratios and incubated overnight prior flow cytometry analysis. Absolute counts for CFSE-negative T cells were quantified. Results were normalized to target only control. Thick lines indicated mean value, error bars indicate standard deviation (n=3).

**Fig. S6: High background in ddPCR assay with probe binding *TRAC* HDRT removed by placing the probe on non-*TRAC* locus of translocation**


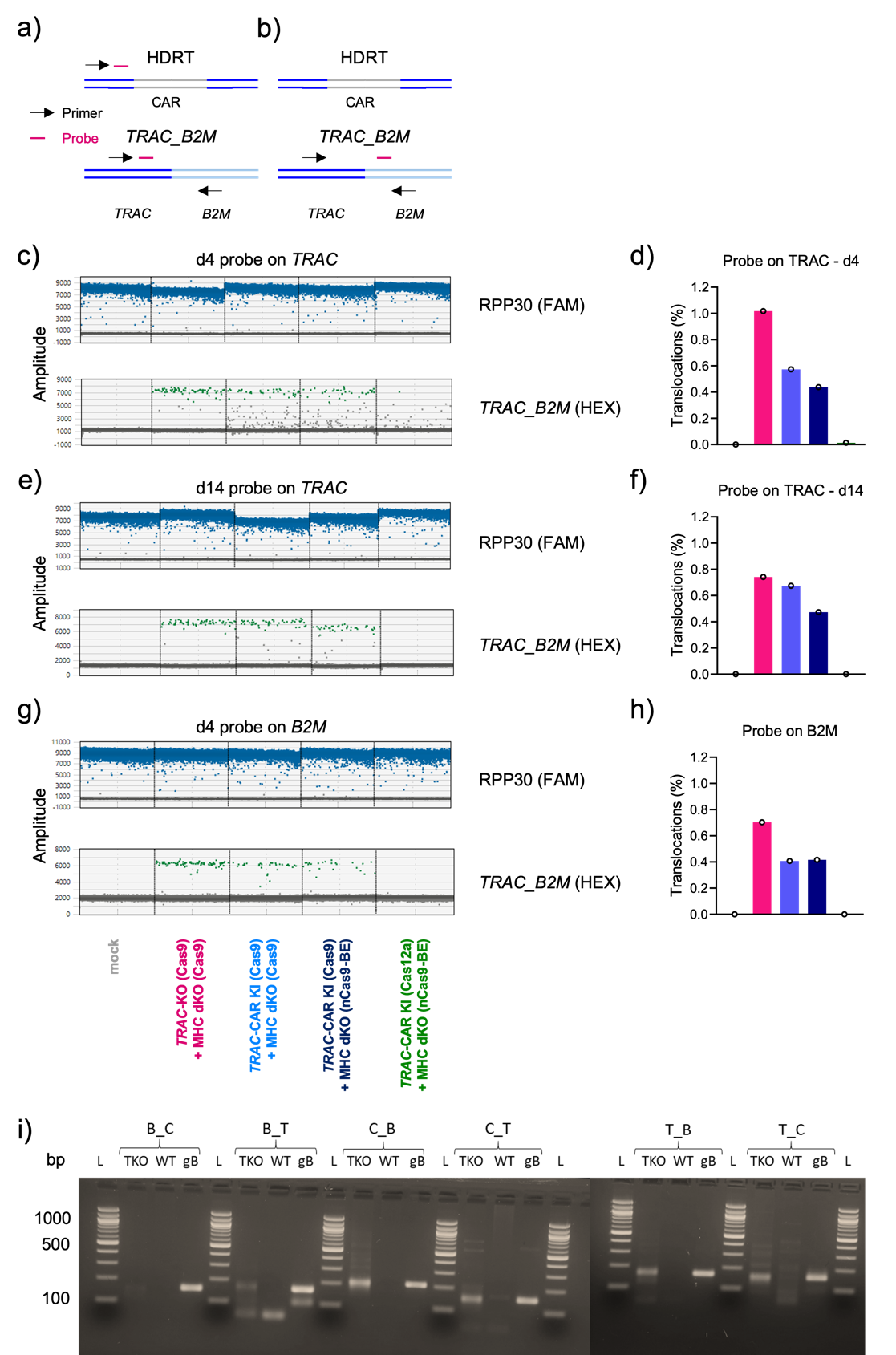


**Fig. S6: High background in ddPCR assay with probe binding TRAC HDRT removed by placing the probe on non-TRAC locus of translocation.** a) ddPCR assay to detect *TRAC_B2M* translocations with the probe binding to the genomic *TRAC* locus as well as to the homology arm of the HDRT. b) Probe binding to B2M prevents binding to HDRT in ddPCR assay detecting *TRAC_B2M* translocations. c) ddPCR raw data using assay described in a) 4 days after gene-editing intervention and d) analysis of different conditions. e) ddPCR raw data using assay described in a) 14 days after gene-editing intervention and f) analysis of different conditions. g) ddPCR raw data using assay described in b) 4 days after gene-editing intervention and h) analysis of different conditions. i) Test PCRs for different ddPCR assays with gene Blocks (gBlocks™, gB) (IDT) as positive control and triple KO cells (TKO).
