# Supplementary material for: Combining different CRISPR nucleases for simultaneous knock-in and base editing prevents translocations in multiplex-edited CAR T cells
Source: Genome Biol. 2023 Apr 24;24:89. doi: 10.1186/s13059-023-02928-7 (PMC10123993; doi:10.1186/s13059-023-02928-7)
Supplement: Supplementary file 5 — Additional file 5. Review history. [file 13059_2023_2928_MOESM5_ESM.docx]

Review History

**First round of review**

**Reviewer 1**

**Are you able to assess all statistics in the manuscript, including the appropriateness of statistical tests used?** No, I do not feel adequately qualified to assess the statistics.

**Comments to author:**

This paper by Glaser et el. explores the possibility of performing multiplex gene editing in primary human T cells to generate off-the-shelf CAR T cells carrying multiple genetic edits. In this setting, the conventional CRISPR/Cas system would fall short due to the high risk of translocations (supported by experimental data in the manuscript) when multiple DNA breaks are induced simultaneously. To mitigate this risk, the authors make use of a nuclease-active Cas enzyme for inserting the CAR gene into the TRAC locus while employing base editors to make single base edits that knock out class I and II genes. Combining this approach takes out the two-way allo-reactivity between donor and recipient cells. This approach to generate allogeneic T cells is as such not novel (see for example PMID 32321775), but the technological method of doing this is elegant and has to my knowledge not been reported before, i.e. combining targeted knock-in using a nuclease-active Cas enzyme with that of base editors. As the authors show, this significantly reduces the number of translocations and constitutes a significant advance in the path for generating safer allogeneic T cell therapies. The experiments are performed in a vigorous manner and the data presented well (with exception of a few minor points below). The conclusions reached are sounds, and I only have minor comments that the authors should address.

Page 2, Line 34: This sentence seems a bit odd. I'm not sure how the proof reading comes into play during a translocation.

Page 2, Line 57: I believe it's worth mentioning that there still have been (at least to my knowledge) no adverse events reported in clinical gene editing trials or in experiments with primary cells in vitro due to translocations, and that the significance of the risk for tumorigenesis is still debatable, particularly when designing sgRNAs carefully.

Page 3, end of introduction. I suggest adding citations that back up the claim or at least suggest that MHC-I and particularly MHC-II knockout is necessary in allogeneic CAR T cells.

Page 3, Line 34. PMID: 32241852 is missing as an important reference for TRAC knock-in.

Several lines have a German sentence that begins with "Fehler....". Please correct all instances.

Headline for Figure 1B should be rephrased to Potential translocations with...

Figures reporting on translocations. I was wondering how the translocations were calculated as '% of cells'. With ddPCR you don't know the distribution of the translocations among the cells. For example, consider two cell equivalents measured by the reference assay (four copies) and two translocations measured (of the same translocation type). The two translocations could be distributed with one translocation in each cell (100% of cells carrying translocations) or both translocations occurring in the same cell (50% of cells carrying translocations). So I suppose translocations can only be reported as something like "% translocations per haploid genome"?

Page 4, line 15. Please explain "base modified " in more detail.

Figure 2+6. Please optimize the color choice since the light and dark blue are difficult to distinguish.

The data in Figure 2 indicating that the sgRNAs are exchanged between Cas9 and BEs is intriguing. I suggest the authors back this claim up by findings in PMID: 30297964.

Figure 4a. Can you elaborate on the HDR donor design. Normally, one would design the SpCas9 donor to have the homology arms split on the cut site, thereby eliminating any need to mutate the PAM to avoid donor cutting. This figure indicates that the intact protospacer+PAM is in the right homology arm?

Figure 4g. It is unclear why the legend below Figure 4g is placed here. I assume it relates to all of c, e, f, and g?

Page 5, Line 16. Please elaborate on this phenomenon on higher HLA-A/B/C levels with Cas12 rather than Cas9. The cells are all still negative as I assume the vertical line denotes a negative/positive gate?

Discussion: One might add PASTE and CAST systems for DSB-free approaches for targeted large-gene knock-in.

Discussion: The missing self-response by NK cells should be introduced in the introduction as skilled immunologists might already wonder why this hasn't been at least textually addressed in the editing approach when trying to remove class I and II.

**Reviewer 2**

**Are you able to assess all statistics in the manuscript, including the appropriateness of statistical tests used?** Yes, and I have assessed the statistics in my report.

**Comments to author:**

SUMMARY

Glaser et al. report a combination of nuclease/HDR mediated knock-in (KI) and base editor (BE) mediated multiplex knock-out (KO) in human T cells with reduced translocation byproducts.

KI of a CD19-CAR into the TRAC locus is accompanied by concurrent KO of B2M and CIITA to generate an allogeneic CAR-T cell product.

The authors describe how using SpCas9 nuclease together with SpCas9-based ABE leads to gRNA swapping with persisting (albeit reduced) translocations.

They overcome this problem by using an AsCas12a variant for HDR-mediated KI together with a SpCas9-based ABE variant.

This final constellation enables therapeutically relevant (and increased) CAR KI rates of ~30% (similar to lentivirally transduced CAR-T products in clinical use),

efficient ABE-mediated multiplex-KO that prevents NK cell mediated destruction of the allogeneic CAR-T product, and substantially reduced translocation frequencies.

ASSESSMENT

This study is well-executed and the findings are highly relevant.

The combination of different CRISPR editing modalities to yield complex cell products is both elegant and exciting.

This approach enables safety-enhanced cell products with large payload integration (HDR) and multiplex-KO which is relevant to the entire field of gene/cell therapy.

The focus on translocations (and reduction thereof) is important for clinical translation of newer CAR-T therapies, given similar products are in clinical development and multiplex-KO is needed in all allogeneic T cell products.

MINOR

P4, Lines 50-56: I don't understand this case. The study was halted because CAR-T cells showed translocations but the CAR-T product didn't contain cells with translocations?

P5, Lines 5-7 vs. 9-11 are redundant.

P7, Lines 53-54: Why is MHC class I expression in TRAC-CAR KI (Cas12a) + MHC dKO (BE) cells higher (and compared to what)? Sorry, if I missed this.

P9, Lines 17-19: REF for the passage on AAV6-enhanced KI?

Fig. 1f: Could the authors please explain why ~50% of the mock treated cells are single+?

Fig. 1h: Somewhat hard to grasp how mock negative control (grey) vs. TRAC-KO + MHC dKO (pink) is not significantly different? Is this the right statistical test? Pink vs. blue being n.s. makes sense. Wouldn't this mean that there are no significant translocations in triple-DSB cells vs. untreated cells?

Fig. 4c, e, g: Could the authors speculate as to why PAM disruption prevents re-targeting (and increases HDR) with Cas12a but not with Cas9?

**Point-by-point response to peer review for manuscript**

**GBIO-D-22-01703**

“Combining different CRISPR nucleases for simultaneous knock-in and base editing prevents translocations in multiplex-edited CAR T cells”

Authors comment: We would like to thank the reviewers for taking the time to review our paper and the constructive comments to improve the clarity and strength of the presented data. We believe we addressed all issues raised by the reviewers. To this end, we performed additional experiments to repeat the translocation analysis as well as flow cytometry readouts to strengthen our claims in Figure 1, 2 and 5 (by higher n to provide a more valid statistical analysis) and we have introduced changes to text or figures as suggested by the reviewers.

Reviewer #1: This paper by Glaser et el. explores the possibility of performing multiplex gene editing in primary human T cells to generate off-the-shelf CAR T cells carrying multiple genetic edits. In this setting, the conventional CRISPR/Cas system would fall short due to the high risk of translocations (supported by experimental data in the manuscript) when multiple DNA breaks are induced simultaneously. To mitigate this risk, the authors make use of a nuclease-active Cas enzyme for inserting the CAR gene into the TRAC locus while employing base editors to make single base edits that knock out class I and II genes. Combining this approach takes out the two-way allo-reactivity between donor and recipient cells. This approach to generate allogeneic T cells is as such not novel (see for example PMID 32321775), but the technological method of doing this is elegant and has to my knowledge not been reported before, i.e. combining targeted knock-in using a nuclease-active Cas enzyme with that of base editors. As the authors show, this significantly reduces the number of translocations and constitutes a significant advance in the path for generating safer allogeneic T cell therapies. The experiments are performed in a vigorous manner and the data presented well (with exception of a few minor points below). The conclusions reached are sounds, and I only have minor comments that the authors should address.

Author response: We would like to thank the reviewer for his/her time and positive assessment of our work.

Page 2, Line 34: This sentence seems a bit odd. I'm not sure how the proof reading comes into play during a translocation.

Author response: Thanks for pointing this out. We have corrected the wording to the following

“*By design, multiplex editing using conventional CRISPR-Cas nucleases induces multiple DNA DSBs and can result in chromosomal translocations via NHEJ between the different targeted loci.”*

Page 2, Line 57: I believe it's worth mentioning that there still have been (at least to my knowledge) no adverse events reported in clinical gene editing trials or in experiments with primary cells in vitro due to translocations, and that the significance of the risk for tumorigenesis is still debatable, particularly when designing sgRNAs carefully.

Author response: We agree with the reviewer’s point to highlight that no adverse effects or malignant transformations have been linked to translocations associated with gene editing. Further, we are not aware of any incidental transformations after CRISPR-Cas9 gene editing of primary human T cells. However, multiple groups have used gene editing to induce translocations associated with malignancy on purpose to model oncogenesis. One leading example is the study by Reimer et al (Haematologica 2017, PMID: 28572162): The authors used CRISPR-Cas9 to induce the MLL-ENL translocation and thereby initiated leukemia development in primary human CD34+ hematopoietic stem cells after engraftment into an immunodeficient mouse.

We have adjusted the wording to the following:

“*Thus far, translocations have not been linked to adverse effects or malignant transformation in clinical trials employing gene editing. However, the caution exercised by the regulatory authorities highlights that translocations remain a concern in the clinical translation of multiplex gene-edited T cell products and should be avoided.*”

Page 3, end of introduction. I suggest adding citations that back up the claim or at least suggest that MHC-I and particularly MHC-II knockout is necessary in allogeneic CAR T cells.

Author response: We thank the reviewer for this comment. We previously stated that the editing of MHC-I and MHC-II protects from allo-specific CD4+/CD8+ T cell responses. We have adjusted the wording to emphasize that we and others believe this will improve the persistence of the CAR-T cell products. Importantly, while CD8+ T cells are the canonical allo-specific “cytotoxic” T cells, previous studies have demonstrated that a subset of allo-specific CD4+ T cells can also induce cytolysis in mice (PMID: 53265, PMID: 67179) and humans (PMID: 84045). We added citations for one original citation on cytotoxic CD4+ T cells and a recent mini-review on cytotoxic CD4+ T cells.

Page 3, Line 34. PMID: 32241852 is missing as an important reference for TRAC knock-in.

Author response: We thank the reviewer for this important remark. The study by Wiebking et al is in fact an important demonstration of the TRAC-knock-in platform. We have added the reference here and at the second time in the discussion (as an example of very high knock-in rates achieved with AAV6+gene editing).

Several lines have a German sentence that begins with "Fehler....". Please correct all instances.

Author response: We are very sorry for this mistake. The error message was due to faulty hyperlinks to supplementary figures. We corrected this throughout the entire manuscript.

Headline for Figure 1B should be rephrased to Potential translocations with...

Author response: Done.

Figures reporting on translocations. I was wondering how the translocations were calculated as '% of cells'. With ddPCR you don't know the distribution of the translocations among the cells. For example, consider two cell equivalents measured by the reference assay (four copies) and two translocations measured (of the same translocation type). The two translocations could be distributed with one translocation in each cell (100% of cells carrying translocations) or both translocations occurring in the same cell (50% of cells carrying translocations). So I suppose translocations can only be reported as something like "% translocations per haploid genome"?

Author response: We would like to thank the reviewer for this very constructive comment. We agree that “Translocations (% per haploid genome)” is more precise and have corrected the axis labels in all figures related to ddPCR of translocations. Thanks a lot!

Page 4, line 15. Please explain "base modified " in more detail.

Author response: We have added the base modification detail to the text.

*“To this end, we generated in-vitro transcribed, N1-metyhlpseudouridine-base modified mRNA of the adenine base editor ABE8.20m, which is comprised of an evolved version of the TadA deminase enzyme fused to a catalytically impaired Cas9 nickase (nCas9), as previously described (42).”*

Figure 2+6. Please optimize the color choice since the light and dark blue are difficult to distinguish.

Author response: Thanks for pointing this out. We adapted the colors to increase the contrast to make it easier to distinguish the conditions.

The data in Figure 2 indicating that the sgRNAs are exchanged between Cas9 and BEs is intriguing. I suggest the authors back this claim up by findings in PMID: 30297964.

Author response: Thank you very much. We have added the citation which nicely complements our claim here and in the discussion section.

Figure 4a. Can you elaborate on the HDR donor design. Normally, one would design the SpCas9 donor to have the homology arms split on the cut site, thereby eliminating any need to mutate the PAM to avoid donor cutting. This figure indicates that the intact protospacer+PAM is in the right homology arm?

Author response: We understand the reviewer’s surprise. The HDR donor for TRAC knock-in used in this study was originally described by Roth et al 2018 (PMID: 29995861, Addgene ID: Plasmid #112021). In their design, Roth et al did not adhere to the usual “split” design, but only introduced a silent mutation in the PAM of the Cas9-sgRNA recognition sequence which was placed on the right homology arm. In a previous study, we screened for gRNAs for Cas12a knock-ins into the *TRAC* locus (see Figure 3, PMID: 35573047): The combination of the Cas12a gRNA used in this study and the HDR donor from Roth et al 2018 resulted in the best knock-in rates. We later realized that the protospacer+PAM sequence for our Cas12a-gRNA was almost completely intact on the template (intact PAM + 21/23 nucleotides aligned), which may be enough to cut the dsDNA. To our surprise, this still achieved relatively good knock-in rates. Mutating the PAM of Cas12a to prevent binding & cutting of dsDNA HDR template improved the knock-in rates as presented in Figure 3. Therefore, the “non-split” HDR donor template design may be a useful alternative to classical “split” designs.

Figure 4g. It is unclear why the legend below Figure 4g is placed here. I assume it relates to all of c, e, f, and g?

Author response: We agree with the reviewer that the original placement of the legend was confusing and have eliminated it.

Page 5, Line 16. Please elaborate on this phenomenon on higher HLA-A/B/C levels with Cas12 rather than Cas9. The cells are all still negative as I assume the vertical line denotes a negative/positive gate?

Author response: Yes, we believe the cells have heavily downregulated HLA-A/B/C-expression. When base editing is the primary outcome at the B2M target site (Figure 5), the HLA-A/B/C expression is drastically reduced (black line denotes positive). However, MCH-I expression still appears to be relatively higher than when the same site was targeted with a nuclease /indels. Another display of the results can be found in Supplementary Figure 2. The black line always denotes the lower end of the HLA-A/B/C positive population. Everything to the left of the black line signifies strong MHC-I downregulation (log scale). In Supplementary Figure 2, we have also included a red line to highlight that there seem to be two populations in the Cas9-edited populations: one negative HLA-A/B/C population, which is only found in T cells edited with the Cas9 nuclease at the B2M site (presumably complete), and another HLA-A/B/C-low/dim population, which is enriched in the Cas12a + BE samples. Multiple groups have previously demonstrated that splice site editing may not eliminate all protein (Gaudelli et al 2020; Kluesner et al 2021), therefore we attempted to functionally validate whether B2M-splice site editing achieved the intended purpose (protection from allo-recognition). We confirmed that the downregulation observed with Cas12a + BE effectively protects from allo-specific T cell killing to a similar degree as the Cas9-treated conditions. We introduced changes to the wording to clarify this point.

Discussion: One might add PASTE and CAST systems for DSB-free approaches for targeted large-gene knock-in.

Author response: The reviewer makes a great point. We have added references and added a few sentences to the discussion as follows:

“Further, new technologies for site-specific gene delivery without DNA DSBs, such as PASTE ^52^ (which is a combination of CRISPR prime editing^53^ and serine integrases^54^), CRISPR-associated transposases (CAST)^55,56^ or designer recombinases^57^, may allow translocation-free multiplex-editing after optimization for high efficacy in primary human cells. “

Discussion: The missing self-response by NK cells should be introduced in the introduction as skilled immunologists might already wonder why this hasn't been at least textually addressed in the editing approach when trying to remove class I and II.

Author response: We agree with the reviewer and have added this fact in the introduction.

Reviewer #2: SUMMARY

Glaser et al. report a combination of nuclease/HDR mediated knock-in (KI) and base editor (BE) mediated multiplex knock-out (KO) in human T cells with reduced translocation byproducts.

KI of a CD19-CAR into the TRAC locus is accompanied by concurrent KO of B2M and CIITA to generate an allogeneic CAR-T cell product.

The authors describe how using SpCas9 nuclease together with SpCas9-based ABE leads to gRNA swapping with persisting (albeit reduced) translocations.

They overcome this problem by using an AsCas12a variant for HDR-mediated KI together with a SpCas9-based ABE variant.

This final constellation enables therapeutically relevant (and increased) CAR KI rates of ~30% (similar to lentivirally transduced CAR-T products in clinical use),

efficient ABE-mediated multiplex-KO that prevents NK cell mediated destruction of the allogeneic CAR-T product, and substantially reduced translocation frequencies.

ASSESSMENT

This study is well-executed and the findings are highly relevant.

The combination of different CRISPR editing modalities to yield complex cell products is both elegant and exciting.

This approach enables safety-enhanced cell products with large payload integration (HDR) and multiplex-KO which is relevant to the entire field of gene/cell therapy.

The focus on translocations (and reduction thereof) is important for clinical translation of newer CAR-T therapies, given similar products are in clinical development and multiplex-KO is needed in all allogeneic T cell products.

MINOR

P4, Lines 50-56: I don't understand this case. The study was halted because CAR-T cells showed translocations but the CAR-T product didn't contain cells with translocations?

Author response: We understand the reviewer’s confusion. Since we submitted our manuscript, the mentioned case was published by Sasu et al in Molecular Therapy (PMID: 36518079). We modified the section of the text to improve the clarity and accurateness of the findings. The clinical trial was halted because a patient who developed bone marrow aplasia after receiving allogeneic gene-edited CAR T cells. The health care team performed a cytogenetic analysis of the bone marrow and found an inversion at chromosome 14 (which was one of the chromosomes targeted by TALEN gene editing). Genomic analysis revealed that the breakpoints of the inversion did not involve the sites in the TRAC gene which were targeted with the TALE-nuclease. Further, the authors could not detect the inversion in the product prior to infusion. The pattern of the chromosomal abnormality detected in the patients fits to RAG1-mediated rearrangement, which is frequently observed at the TCR alpha chain locus on Chromosome 14. Therefore, the authors speculated whether the observed donor cell clone may have arisen from a recombination event after infusion (a process called TCR revision). Overall, the clone contracted and eventually disappeared without causing any adverse effects and could be considered a co-incidental finding in patient developing an expected adverse event after lymphodepletion chemotherapy and CAR-T cell administration.

The adjusted text states the following:

*“Recently, a clinical trial was halted due to the detection of CAR T cells bearing a chromosomal translocations in a patient with who developed bone marrow aplasia after he received allogeneic multiplex TALEN-edited CAR T cell product (#NCT04416984; clinicaltrials.gov) (REF 25). The trial was later continued, because the chromosomal abnormality did not involve the sites targeted by TALEN gene editing nor was it found in the edited product prior to infusion (REF 25). Thus far, translocations induced by gene editing have not been linked to adverse effects or malignant transformation in clinical trials employing gene editing. However, the caution exercised by the regulatory authorities highlights that translocations remain a concern in the clinical translation of multiplex gene-edited T cell products and should be avoided.”*

P5, Lines 5-7 vs. 9-11 are redundant.

Author response: We thank the reviewer for his comment and have eliminated the duplicated wording in the mentioned lines.

P7, Lines 53-54: Why is MHC class I expression in TRAC-CAR KI (Cas12a) + MHC dKO (BE) cells higher (and compared to what)? Sorry, if I missed this.

Author response: We believe this may be related to different gene editing outcome at the B2M side after base editing vs regular Cas9 cutting, which results in different indels and different degree of protein loss. There seem to be two populations in the Cas9-edited populations- one negative HLA-A/B/C population, which is only found in T cells edited with the Cas9 nuclease at the B2M site (Suppl. Fig. 2, left to red line indicates complete protein loss), and another HLA-A/B/C-low/dim population, which is enriched in the Cas12a + BE samples and may indicate a little residual protein. As T cells can detect as little as 1 antigen-MHC molecule on a target cell surface with their TCR, we tested whether the MHC-I dim population actually is functional (e.g. regularly expressed MHC-I). Our allo-specific T cells could not lyse either tKO, dKO (Cas9) or dKO (Cas12a+BE) conditions. Therefore, we conclude the MHC-I downmodulation achieved by base editing the B2M splice sites is enough prevent recognition from allo-specific T cells.

P9, Lines 17-19: REF for the passage on AAV6-enhanced KI?

Author response: We have added a references exemplifying high degree of KI in T cells using AAV and one additional reference for the mechanisms underlying AAV transduction. We also slightly modified the wording of the sentence to the following:

“*AAV6’s high efficacy for KI (Ref) may be explained by the virus-mediated delivery of templates to the cells’ nuclei (Ref), thereby increasing the probability for HDR-mediated KI due to the high local density of repair templates*.”

Fig. 1f: Could the authors please explain why ~50% of the mock treated cells are single+?

Author response: We apologize for the lack of clarity in the figure. In fact, ca. 50% of the mock-treated cells are douple+, because they express CD3 and HLA-I, but not HLA-II. To improve the clarity, we have revised the coloring of Figure 1f.

Fig. 1h: Somewhat hard to grasp how mock negative control (grey) vs. TRAC-KO + MHC dKO (pink) is not significantly different? Is this the right statistical test? Pink vs. blue being n.s. makes sense. Wouldn't this mean that there are no significant translocations in triple-DSB cells vs. untreated cells?

Author response: We agree with the reviewer’s impression. The lack of statistical significance was due to the low sample size (n=3) and the correction for multiple testing in the one-way ANOVA which we used for statistical testing. We have repeated the experiment and performed ddPCR analysis for translocations in two additional healthy donors and the updated statistics in Figures 1,2 and 5, accordingly. The results are in line with the previous claims and support the reviewer’s impression of significance.

Fig. 4c, e, g: Could the authors speculate as to why PAM disruption prevents re-targeting (and increases HDR) with Cas12a but not with Cas9?

Author response: The introduction of the mutation in the Cas12a PAM was outside of the Cas9-gRNAs recognition sequence; therefore, it should only have minor impact on HDR when employing the Cas9-gRNA RNP. While our Cas12a gRNA was matched in 21 of 23 nucleotides of the gRNA, it’s PAM (purple below) was originally intact. The additional mutation which we introduced into the Cas12a gRNA’s PAM does not overlay with the Cas9-gRNA (see below).

**Second round of review**

**Reviewer 1**

The authors has satisfactorily addressed all my points. Congratulations on the great work.
